# The relationship between regular tea drinking and calcification of the coronary arteries

**DOI:** 10.34172/jcvtr.2022.12

**Published:** 2022-06-21

**Authors:** Amir Reza Sajjadieh khajouui, Jamshid Najafian, Reza Talebzadeh, Majid Nejati, Mohaddeseh Behjati

**Affiliations:** ^1^Department of Internal Medicine, Isfahan University of Medical Sciences, Isfahan, Iran; ^2^Cardiovascular Research Center, Cardiovascular Research Institute, Isfahan University of Medical Sciences, Isfahan, Iran; ^3^Al-Zahra Hospital, Isfahan University of Medical Sciences, Isfahan, Iran; ^4^Anatomical Sciences Research Center, Institute for Basic Sciences, Kashan University of Medical Sciences, Kashan, Iran; ^5^Hypertension Research Center, Cardiovascular Research Institute, Isfahan University of Medical Sciences, Isfahan, Iran

**Keywords:** Coronary Artery, Calcification, Black Tea, Atherosclerosis

## Abstract

***Introduction:*** Coronary Atherosclerosis is the leading cause of death and disability worldwide. Atherosclerosis could be detected noninvasively by coronary calcification, measured by calcium score in CT angiography. Dietary factors are influential in the evolution of coronary plaques, and one of the most prevalent drinks is black tea. We aimed to evaluate the effects of black tea on coronary calcium scores.

***Methods:*** This cross-sectional analytical descriptive study was conducted on 200 candidates for CT angiography referred by their physician because their symptoms were suggestive of ischemia. A questionnaire was filled out for every participant, and the habit of tea drinking was asked and marked as none drinker, 1-3 cups per day and >3 cups per day.

***Results:*** 89.5% of the participants consumed tea. The mean calcium score in patients who did not drink tea was 674.9±154.74 in those patients who drank 1-3 glasses per day, 269.5±46.9 and in those who drank more than three glasses of tea and was 261.1±45.2. There was a significant statistical relationship between calcium scores and tea intake, independent to other traditional risk factors (*P*= 0.001). Significant coronary artery plaques were also less prevalent in those who drank tea (36% and 41% in 1-3 and >3 cups, respectively) than non-drinkers (67%). Still, the number of involved vessels was not significantly different.

***Conclusion:*** Regular black tea consumption could have protective effects on coronary artery calcification.

## Introduction

 Apart from water, tea is one of the most widely consumed beverages worldwide.^1,2^ Both black and green tea are prepared from a shrub called Camellia Sinensis, with different processing methods. Black tea is fermented before the last stage of heating^2^. Black and green tea have many health benefits, including healthy bones, antioxidants properties, oral health, better heart rate, healthy digestive tract, decreased stress level, and happiness factor.^3-5^

 Drinking tea has been correlated with a low incidence of cardiovascular (CV) diseases and cancer, in which oxidative stress plays a critical role. Catechin and flavin are two bioactive phytochemicals responsible for the antioxidant activity of green and black tea.^6-8^

 CV calcification is a systemic disease that is considered an independent predictor of CV events and all-cause mortality and or renal diseases.^9^ Coronary artery calcification (CAC) scoring provides improved predictive ability over conventional risk factor scoring alone.^10-13^ The initiation of CV calcification resembles the process of osteogenesis. Studies have shown that osteoblast- and osteoclast-like cells and bone matrix proteins resulted in hydroxyapatite (calcium phosphate) deposition in the arterial wall. In the case of more severe calcification, fully formed bone could be observed in arteries and valves.^14,15^ There is no specific treatment for arterial calcification, and suggested medications such as statins, vasodilators, and other therapies for Atherosclerosis have negligible effect. Thus, it would be better to apply mechanisms such as anti-oxidation to prevent vascular calcifications.^16^ There is some evidence that moderate tea drinkers lead to slower progression of CAC deposition with subsequent reduced risk for CV events.^17^ Due to the controversies, this study aimed to evaluate the association between tea drinking and CAC score.

## Materials and Methods

 This cross-sectional analytical descriptive study was approved by the ethics committee of Isfahan University of Medical Sciences (code: 3964002) and conducted in Al-Zahra hospital in Isfahan city from April 2015 to March 2018. Participants were selected from patients referred for computed tomography (CT) angiography to evaluate coronary artery disease (CAD). The patients without contraindication for CT angiography were included in this study. However, patients with a medical history of coronary artery interventions, cardiac surgery, congenital heart disease, inherited metabolic diseases, and renal failure were excluded from this study. Two hundred patients were enrolled in this study after getting signed informed consent. Demographic characteristics of the patient, including age, gender, and occupation, were recorded. Blood pressure, height, and weight of the patients were measured, too. Medical history of CV diseases, hypertension, diabetes, and some of its risk factors such as smoking and hyperlipidemia were checked. Patients were evaluated for tea-drinking habits using a questionnaire developed at the University of Arizona followed by translation and validation^4^. It was initially addressed by 20 patients to determine its reliability. The existing deficiencies were solved, and Cronbach’s alpha was calculated as 0.88 in the preliminary survey.

 CT angiography was done with a standard protocol with GE system, and analysis was done using ADW4.4 software. Each patient’s calcium scores (CS) were determined and recorded in each coronary artery. CS was divided into 5 groups including normal group (CS = 0), minimal (CS = 1-10), mild (CS = 11- 100), moderate (CS = 101-400) and severe (CS> 400). Atherosclerotic plaques were determined and the number of arteries with significant and non-significant stenosis were also determined and recorded.

###  Statistical analysis

 The required sample size for this study was derived from the formula considering the 95% confidence level (Z_1-α/2_ = 1.96), the prevalence of coronary calcification in tea consumers due to the lack of a similar study. Internal was considered a rate of 0.5, and the acceptance of an error rate of 0.075 was estimated at 170 people. The sample size was calculated by the following formula: $n=\left[{\left(Z_{1-\alpha /2}\right)}^{2}\times P\left(1-P\right)\right]\;/d^{2};\;n=\left[{\left(1.96\right)}^{2}\times 0.5\left(1-0.5\right)\right]\;/{\left(0.075\right)}^{2}$ . For more assurance, 200 patients were studied.

 Data analysis was done by SPSS version 24 software. Descriptive statistics were presented by mean ( ± standard deviation) and frequency (percentage). Kolmogorov-Smirnov test before analysis was done for determining data normality. The Chi-square test compared qualitative data between the case and control groups. Comparison of quantitative variables was made by independent sample T-test and One-way ANOVA. Considering the abnormality of CA distribution, the data were presented in median (25-75) (IQR) and analyzed by non-parametric Mann Whitney and Kruskal Wallis tests. Logistic regression test using backward conditional method was used to determine the effect (odds ratio) of variables affecting calcium score, eliminate the effect of confounding variables, and adjust the effect of variables. Statistically significant data was considered at *P* value <0.05.

## Results

**Figure 1:**
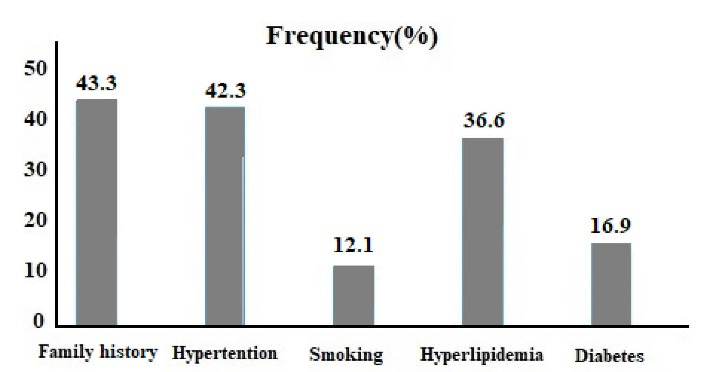
frequency percentage of risk factors in studied patients

 In this study, two hundred patients who underwent CT angiography aged 58.3 ± 11.5 (ranging from 30 to 83) participated. 108 (54%) were male, and 92 (46%) were female. Zero, 1, 2, 3, and 4 traditional risk factors for CAD were detected in 32, 70, 58, 30, and 10 patients, respectively. The percentage of CV risk factors is shown in Figure 1. Totally, 179 patients (89.5%) consumed tea. The average amount of tea intake was 3.27 ± 1.9 glasses (ranges from 0 to 10 glasses), equal to 816 ± 478 ml/day. Of 200 enrolled subjects, 164 (82%) were tea consumers. Among them, 86 (43%) and 78 (39%) consumed more than three glasses of tea and between 1-3 glasses per day, respectively. Table 1 shows the distribution of tea drinking in terms of demographic characteristics and risk factors for CV disease. According to the table, there was no significant difference in tea consumption and tea drinking by age, sex, occupation, and CV risk factors.

**Table 1:** Frequency of demographic characteristics and cardiac risk factors in terms of tea intake

**Variables**		**Black tea consumption**			**Amount of Black tea consumption**			
		**Yes (164)**	**No (36)**	***P***	**No**	**1-3 glasses**	**>3 glasses**	***P***
Age mean ± SD (year)		57.46 ± 11.6	62.36 ± 9.97	0.02	62.4 ± 10	59.2 ± 11.9	55.8 ± 11.3	0.011
Sex N (%)	Male	86(52.4)	22(61.1)	0.34	22(61.1)	42(53.8)	44(51.2)	0.6
	Female	78(47.6)	14(38.9)		14(38.9)	36(46.2)	42(48.8)	
Occupation	Worker	9(5.5)	4(11.1)	0.55	4(11.1)	3(3.8)	6(7)	0.81
	Employee	25(15.2)	6(16.7)		6(16.7)	11(14.1)	14(16.3)	
	Free job	43(26.2)	6(16.7)		6(16.7)	23(29.5)	20(23.3)	
	Household	67(40.9)	14(38.9)		14(38.9)	31(39.7)	36(41.9)	
	Retired	20(12.2)	6(16.7)		6(16.7)	10(12.8)	10(11.6)	
Cardiovascular risk factors	Family history	74(45.1)	14(38.9)	0.5	14(38.9)	38(48.7)	36(41.9)	0.54
	Hypertension	72(43.9)	16(44.4)	0.95	16(44.4)	32(41)	40(46.5)	0.78
	Smoking	19(11.6)	8(22.2)	0.091	8(22.2)	8(10.3)	11(12.8)	0.21
	Hyperlipidemia	60(36.6)	18(50)	0.14	18(50)	27(34.6)	33(38.4)	0.29
	Diabetes	26(15.9)	9(25)	0.19	9(25)	9(11.5)	17(19.8)	0.16

**Figure 2:**
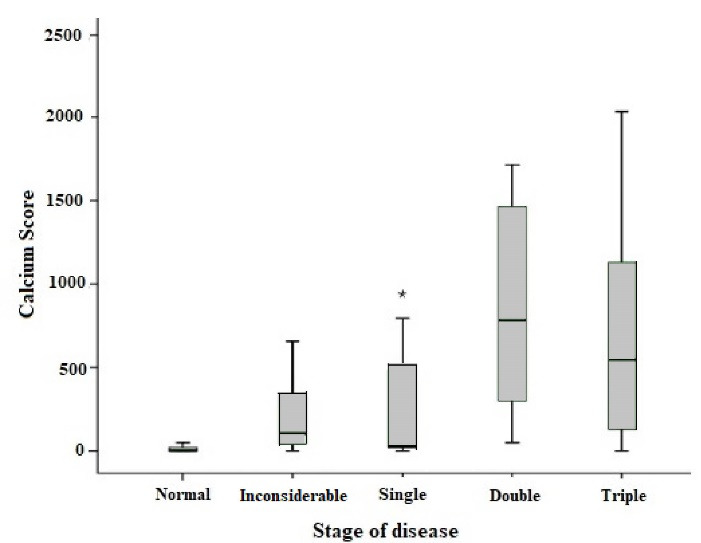
Median, range and 25-75 percentile of calcium score based on disease stage

 The median calcium score among participants was 67 (12-356). The percentage of CS in patients is shown in Figure 2. The correlation test showed a reverse correlation (-0.16) between CAC scores and the amount of tea consumption (*P* = 0.036). According to the findings of CT angiography, CA status in 111(55.5%) was normal (CA≤100), and in 89(44.5%) was abnormal (CA>100). The mean CAC score was 674.9 ± 154.74, 269.5 ± 46.9, and 261.1 ± 45.2 in 0, 1-3, and >3 glasses tea drinkers, respectively.

 This result indicated a significant relationship between CAC scores and tea intake (*P*< 0.001). The distribution of demographic variables, CV risk factors, and tea intake based on CAC scores are shown in Table 2. CAC scores were significantly different in age, tea consumption, high blood pressure, smoking, and hyperlipidemia.

**Table 2:** Distribution of demographic variables, cardiovascular risk factors and tea intake based on calcium scores

**Variables**		**Calcium score**	***P***
Age	<50	87.7 ± 28	<0.001
	≥ 50	379.6 ± 42.9	
Sex	Male	292.2 ± 43.2	0.62
	Female	326.7 ± 54.8	
Occupation	Worker	253 ± 129.5	0.87
	Employer	230.5 ± 63.4	
	Free job	325.8 ± 69.3	
	Household	331.4 ± 60.5	
	Retired	321.9 ± 88.3	
Tea consumption (glasses)	No	674.9 ± 154.7	0.001
	1-3	269.5 ± 46.9	
	≥ 3	261.1 ± 45.2	
Family history	Yes	314.8 ± 47.5	0.82
	No	299.4 ± 49.4	
Hypertension	Yes	252.4 ± 41.6	0.07
	No	378.9 ± 56.5	
Smoking	Yes	635.8 ± 147.1	<0.001
	No	256.9 ± 30.8	
Hyperlipidemia	Yes	451.1 ± 69.1	<0.001
	No	216.6 ± 32.4	
Diabetes	Yes	414.9 ± 98.4	0.08
	No	263.7 ± 33.5	

**Figure 3:**
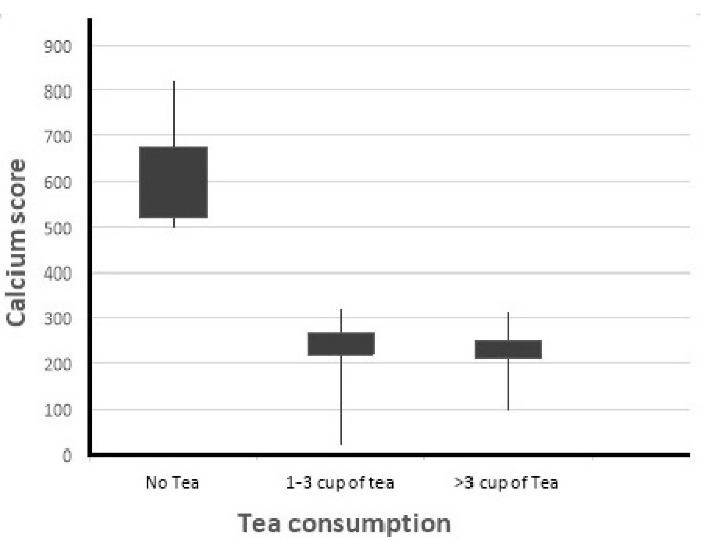
Coronary artery calcium score and tea consumption

 According to the results of CT angiography, 61 (30.5%) were normal, 56 (28%) had non-significant plaques, 22 (11%) had stenosis in one vessel, 16 (8%) had stenosis in two vessels, and 45 patients (22.5%) had stenosis in three coronary arteries. Mean scores of calcium in the five groups were 9.9 ± 1.6, 167.7 ± 21.17, 278.1 ± 107.66, 849.9 ± 158, and 708.98 ± 88.5, respectively, and there was a significant relationship between CAD and calcium score (*P* < 0.001). The median, range, and percentile of 25- 75% CS are shown in terms of the severity of CAD (Figure 3). In terms of tea consumption, the severity of CAD (one, two, or three affected arteries) was not significant. Still, significant coronary artery plaques compared to non-significant plaques were more frequent in those who did not drink tea. The relationship between the number of affected coronary arteries and tea consumption is shown in the Table 3.

**Table 3:** Frequency of disease stage in terms of tea consumption

**Disease Stage**		**Tea consumption**			***P***
		**No**	**1-3 glasses**	**> 3 glasses**	
Number of affected vessels (%)	Normal	5(23.8)	27(32.1)	29(30.5)	0.16
	Inconsiderable	2(9.5)	27(32.1)	27(28.4)	
	One vessel	1(4.8)	9(10.7)	12(12.6)	
	Two vessel	4(19)	5(6)	7(7.4)	
	Three vessel	9(42.9)	16( 19)	20(21.1)	
Coronary artery disease	Non-significant	7(33.3)	54(64.3)	56(58.9)	0.037
	Significant	14(66.7)	30(35.7)	39(41.1)	

 Data analysis using logistic regression through inter method showed that non-consumption of tea enhanced CAC to 4.65 times that was statistically significant (*P* = 0.004). Indeed, age more than 50 years old was associated with an enhanced chance of abnormal CA score to 3.73 times, but other variables had no significant impact on alteration of CA score. On the other way, data analysis using conditional logistic regression through backward conditional method showed that the two mentioned variables had a significant impact on having the chance of normal CA score. In contrast, other variables had no significant confounding effect using both applied methods. Thus, considering the unchanged odds ratio measures (OR), there was no significant confounding impact from other variables such as age and tea consumption (Table 4).

**Table 4:** Effect of demographic variables, cardiovascular risk factors and tea intake on calcium scores

**Variables**		**Calcium score**		Univariate logistic regression*		Multivariate logistic regression**	
		**Normal**	**Abnormal**	**OR(95 % CI)**	***P***	**OR(95 % CI)**	***P***
Tea consumption	Yes	102(91.9)	77(86.5)	4.65(1.66-13.06	0.004	4.36(1.84-10.36)	0.001
	no	9(8.1)	12(13.5)				
Amount of tea consumption	No	9(8.1)	12(13.5)	1.11(0.63-1.95)	0.7	-	-
	1-3 glasses	45(40.5)	39(43.8)				
	≥ 3 glasses	57(51.4)	38(42.7)				
Age	<50	39(35.1)	10(11.2)	3.73(1.62-8.61)	0.002	3.57(1.62-7.87)	0.002
	≥ 50	72(64.9)	79(88.8)				
Sex	Female	52(46.8)	40(44.9)	1.25(0.58-2.65	0.57	-	-
	Male	59(53.2)	49(55.1)				
Occupation	Worker	9(8.1)	4(4.5)	0.98(0.71-1.36	0.91	-	-
	Employer	19(17.1)	12(13.5)				
	Free job	23(20.7)	26(29.2)				
	Household	47(42.3)	34(38.2)				
	Retired	13(11.7)	13(14.6)				
Family history	No	61(55)	51(57.3)	1.04(0.55-1.98)	0.90	-	-
	yes	50(45)	38(42.7)				
Hypertension	No	69(62.2)	43(48.3)	1.84(0.93-3.62)	0.079	1.79(0.97-3.33)	0.064
	Yes	42(37.8)	46(51.7)				
Smoking	No	101(91)	72(80.9)	2.04(0.79-5.3)	0.14	2.26(0.9-5.67)	0.082
	Yes	10(9)	17(19.1)				
Hyperlipidemia	No	74(66.7)	48(53.9)	1.26(0.65-2.43)	0.5	-	-
	Yes	37(33.3)	41(46.1)				
Diabetes	No	93(82.8)	72(80.9)	0.86(0.37-2)	0.72	-	-
	Yes	18(16.2)	17(19.1)				

*Using Logistic regression by Inter method. ** Using Logistic regression by backward conditional method

## Discussion

 The main finding of our study was a reverse correlation between moderate black tea consumption and CAC score. The CAC score in moderate tea drinkers was 22% lower than patients who drink less tea per day. Some studies suggest an inverse relationship between tea consumption and CV diseases.^18-20^ In a survey of 805 men, intakes of tea, onions, and apples were inversely related to CV mortality.^17^ Another follow-up study on 4807 persons for 5.6 years showed an inverse association between tea and flavonoid intakes and myocardial infarction.^21^ Sesso et al demonstrated a lower risk of myocardial infarction in tea consumers than caffeinated and decaffeinated coffee drinkers.^22^ It has also been suggested that regular tea drinkers were associated with a lower prevalence of CAC and lower incidence of subsequent CV events.^19^

 Oxidative stress has been implicated in the development of vascular injury and atherogenesis.^20^ Epidemiological studies have reported a reduced risk of atherosclerosis in people who consume large amounts of flavonoids.^22,23^ Tea contains various active polyphenolic flavonoids called catechins, which make up about 30% of the dry weight of its leaves.^22,24^ These catechins include epicatechin, epicatechin-3-gallate, epigallocatechin, and epigallocatechin-3-gallate (EGCG) as their principal constituent and a well-characterized antioxidant agent. The protective impacts of flavonoids are mostly due to their anti-oxidative activity. Quercetin can inhibit the oxidative changes of low-density lipoproteins that lead to the formation of fatty plaques.^22^ Black tea possesses mainly theaflavins and thearubigins which are known as products of tea catechins. There are various pharmacological properties attributed to these catechins including antioxidative, anti-inflammatory and hypolipidemic effects.^20^

 Some studies have also shown that catechins have effects in preventing and treating cancers. Tea catechins have several effects on cell growth signals. Green tea polyphenols can alter angiogenesis-related miRNA expression in cancers.^25^ Green tea extracts generally showed stronger antioxidant activity than the semi-fermented and black tea extracts.^18^ Polyphenols in green tea have a cardioprotective effect for acute myocardial infraction through free radical-scavenging and antioxidant properties. In vitro studies have shown that polyphenols act as metal ions chelator, ROS or RNS scavenger, ROS generating enzymes inhibitor, and antioxidant enzymes activator.^26^ These compounds in green tea also promote inhibition of NFκb and activation of PI3K/Akt signaling, decrease in expression of Fas receptor, and modulate lipid metabolism, mitochondrial function. All of these and some others like reduction of cytosolic Ca^2+^ overload and sensitivity of myofilament to Ca^2+^ are possible cardioprotective mechanisms of these compounds.^27-29^ In an experimental animal study on a hamster model of Atherosclerosis, both teas were equally effective in inhibiting the development of Atherosclerosis through prevention of hyperlipidemia, antioxidant and anti-fibrinolytic properties.^19^ Endothelial cell dysfunction is involved in the pathogenesis of Atherosclerosis and could be reversed by antioxidant treatment using short- and long-term black tea consumption.^23,30,31^ This finding may partly explain the association between tea intake and decreased CV events.^32^ In addition, studies have shown that daily intake of black tea for one month improves platelet function and reduces platelet activation with subsequent reduction of the risk of atherosclerosis.^33,34^ Reducing the total homocysteine concentration with regular tea consumption is another mechanism for CV protection.^35^

 However, this study did not show a relationship between tea consumption and the number of obstructed coronary arteries and atherosclerotic lesions. It can be argued that many factors contribute to the development of coronary artery disease. Indeed, an enhanced CA score does not necessarily indicate increased stenosis of coronary arteries.^36^ The limitations of this study are the lack of prospective data from tea consumers about clinical events, limited population size (especially in the control group, since most Iranians are accustomed to drinking tea, and people who do not drink black tea at all are rarely found). There was also a lack of information from dietary habits that could affect the CAD prevalence.

## Conclusion

 Black or green tea may positively impact the damage induced by coronary artery calcification as a marker of Atherosclerosis. Possible mechanisms for this effect include reducing platelet activation and direct prevention of calcification.

## Acknowledgments

 The authors thank Al-Zahra Hospital staff for their cooperation in implementing the study.

## Funding

 No financial support has been received from any organization for this work.

## Ethical Approval

 The implementation process of this study was reviewed by the ethics committee of Isfahan University of Medical Sciences and approved by code: IR.MUI.REC.1396.3.004.

## Competing Interest

 The authors state that there is no conflict or competing interest.
